# Functional significance of the signal transduction pathways Akt and Erk in ovarian follicles: *in vitro* and *in vivo* studies in cattle and sheep

**DOI:** 10.1186/1757-2215-1-2

**Published:** 2008-10-01

**Authors:** Kate E Ryan, Claire Glister, Pat Lonergan, Finian Martin, Phil G Knight, Alexander CO Evans

**Affiliations:** 1 School of Agriculture Food Science and Veterinary Medicine, Conway Institute, College of Life Science, University College Dublin, Belfield, Dublin 4, Ireland; 2 School of Biomolecular and Biomedical Science, Conway Institute, College of Life Science, University College Dublin, Belfield, Dublin 4, Ireland; 3 School of Biological Sciences, University of Reading, Whiteknights, Reading, RG6 6AJ, UK

## Abstract

**Background:**

The intracellular signalling mechanisms that regulate ovarian follicle development are unclear; however, we have recently shown differences in the Akt and Erk signalling pathways in dominant compared to subordinate follicles. The aim of this study was to investigate the effects of inhibiting Akt and Erk phosphorylation on IGF- and gonadotropin- stimulated granulosa and theca cell function *in vitro*, and on follicle development *in vivo*.

**Methods:**

Bovine granulosa and theca cells were cultured for six days and stimulated with FSH and/or IGF, or LH in combination with PD98059 (Erk inhibitor) and/or LY294002 (Akt inhibitor) and their effect on cell number and hormone secretion (estradiol, activin-A, inhibin-A, follistatin, progesterone and androstenedione) determined. In addition, ovarian follicles were treated *in vivo* with PD98059 and/or LY294002 in ewes on Day 3 of the cycle and follicles were recovered 48 hours later.

**Results:**

We have shown that gonadotropin- and IGF-stimulated hormone production by granulosa and theca cells is reduced by treatment with PD98059 and LY294002 *in vitro*. Furthermore, treatment with PD98059 and LY294002 reduced follicle growth and oestradiol production *in vivo*.

**Conclusion:**

These results demonstrate an important functional role for the Akt and Erk signalling pathways in follicle function, growth and development.

## Introduction

Folliculogenesis is a vigorously controlled process that involves both proliferation and differentiation of both granulosa and theca cells. These coordinated processes are controlled by local and systemic regulatory factors. The gonadotropins, FSH and LH, are essential for the development of follicles beyond the early antral stage. In both cattle and sheep, ovarian antral follicle growth occurs in a wave-like pattern with 2 to 3 waves per cycle in cattle and 3 to 4 waves in sheep [1]. Wave emergence is triggered by a transient rise in circulating FSH concentrations [2-4], which promotes significant growth of granulosa cells by regulating cell cycle proteins and increasing oestradiol production and the expression of LH receptors [5].

As follicles mature, the largest follicles in the cohort produce high levels of oestradiol and inhibins [6]. This inhibits FSH secretion and the drop in FSH concentrations initiates atresia and regression of the small (subordinate) follicles, whilst the largest (dominant) follicle switches its dependence from FSH to LH and thus avoids regression [7]. FSH and LH exert their stimulatory effect on proliferation and steroidogenesis by binding to specific G protein-coupled receptors which in turn causes an increase in cAMP production and activation of the PKA pathway [8]. While the PKA/cAMP transduction pathway is generally considered to be the primary mediator of gonadotropin action, these hormones also activate other signalling pathways that include activation of the Erk pathway [9,10], the Akt pathway [11,12] and the inositol triphosphate and diacylglycerol [13,14] pathways. These signal transduction pathways, when activated, induce changes in protein activity and gene expression [15]. It is the differential regulation of these pathways and the potential for cross talk between the pathways that is important in mediating the effects of these hormones.

In addition to the gonadotropins, there are numerous growth factors and intraovarian regulators of follicle development and function that include insulin-like growth factor (IGF) and members of the TGF-β superfamily (eg. inhibin-A and activin-A). It has been established that IGF stimulates proliferation of granulosa and theca cells, and enhances the ability of gonadotropins to stimulate steroidogenesis in both granulosa and theca cells [16-18]. In addition, it has been shown that IGF has a direct anti-apoptotic effect and is selectively expressed in healthy follicles compared with small atretic follicles [19]. The Akt and Erk pathways are considered the principle signalling pathways that mediate the effects of IGF [20].

We have previously shown higher levels of total and phosphorylated Akt and Erk in dominant follicles compared with subordinate follicles [21,22]. The objectives of the studies reported here were to examine the interactions of the gonadotrophins and IGF with the Akt and Erk signalling pathways in theca and granulosa cells *in vitro* and to describe their functional significance for ovarian follicle growth *in vivo*.

## Materials and methods

### Experimental design

#### Experiment 1

The aim was to test the hypothesis that FSH and IGF activate Akt and Erk pathways in bovine granulosa cells cultured *in vitro*. This was done using granulosa cells collected from 4 to 6 mm follicles from animals after slaughter using a validated granulosa cell culture system that maintains FSH responsiveness, oestradiol secretion and minimizes luteinization [23]. Granulosa cells were cultured (see below) in serum-free conditions for 144 h with conditioned medium collected and replaced with fresh media (McCoy's 5A modified medium supplemented with 1% (v/v) antibiotic/antimycotic solution, 10 ng/ml bovine insulin, 2 mM L-glutamine, 10 mM HEPES, 5 μg/ml apotransferrin, 5 ng/ml sodium selenite, 0.1% BSA and 10^-7^M androstenedione (all purchased from Sigma)) +/- treatments every 48 hours as described by Glister et al [23]. Cells were seeded at a density of 0.5 × 10^6 ^viable cells per well in 24 well plates and cultured in a 1 ml volume of media +/- treatments. Treatment groups were as follows (i) untreated controls, (ii) 0.33 ng/ml FSH (oFSH-19SIAPP, NIDDK), (iii) 10 ng/ml IGF (recombinant IGF-I analogue, LR3 IGF-I, Sigma, Dublin, Ireland), (iv) 0.33 ng/ml FSH and 10 ng/ml IGF. These treatments (and dose-levels) have been shown previously to stimulate cell proliferation/survival and hormone secretion by bovine granulosa cells over a 144 h treatment period [23]. The more potent LR3 IGF-I analogue was used rather than IGF-I or IGF-II because its action is not compromised by association with endogenous IGF-BPs produced by the cells [24]. At the end of culture, conditioned media were collected and stored at -20°C until assayed for oestradiol, progesterone, inhibin-A, activin-A and follistatin. Cells were scraped off the culture plates in 1 ml of phosphate-buffered saline and a small (50 μl) aliquot of cell suspension was taken and processed for viable cell number by neutral red dye uptake as described previously [23]. The remaining cell suspension was spun at 800 g and the cell pellet washed twice before snap freezing the cell pellet and storing at -80°C until processed for Western blots. Western blot analysis was used to determine the levels of Akt and Erk and their phosphorylated proteins p-Akt and p-Erk in total protein extracted from cells at the end of culture (see below).

The experiment was done on 4 separate occasions (replicates) with 6 wells included per treatment per replicate.

#### Experiment 2

The aim was to test the hypothesis that pharmacological inhibition of the activation of the Akt and Erk pathways would inhibit the actions of FSH and IGF on bovine granulosa cells *in vitro*. Granulosa cells were cultured as described above with one of four possible culture media; control medium, FSH (0.33 ng/ml), IGF (10 ng/ml) or FSH plus IGF in combination. Additionally each of the above treatments was given in combination with either PD98059 (513000, Calbiochem, VWR International Ltd., Ashbourne, County Meath, Ireland), a specific inhibitor of the Erk activating enzyme MEK (APK/Erk kinase) [25] or LY294002 (L9908, Sigma, Dublin, Ireland), a specific inhibitor of Akt activation [26] or a combination of both inhibitors resulting in a total of 16 treatments. Both PD98059 and LY294002 were initially dissolved in DMSO and were diluted to a final concentration of 50 μM *in vitro*. Control media also contained DMSO at a final concentration of 0.005% (v/v) in all treatment groups.

#### Experiment 3

Theca interna cells were isolated from the same sets of follicles used in experiment 2 as described by Glister et al [26]. Theca cells were plated out and cultured using the same serum-free conditions as described above for granulosa cells except that androstenedione was omitted from the culture medium. Cells were cultured for 144 h with control media, media with LH (160 ng/ml, oLH-S26, NIDDK) and the same treatments in combination with PD98059 (50 μM) and/or LY294002 (50 μM). The dose-level of LH used here was shown previously to promote optimal secretion of androstenedione by bovine theca cells cultured under these conditions [26]. Media were changed and treatments replenished every 48 h. At the end of culture, conditioned media were collected and stored at -20°C until assayed for androstenedione and progesterone. Viable cell number was determined by neutral red dye uptake. The experiment was done on 4 separate occasions (replicates) with 6 wells included per treatment per replicate.

#### Experiment 4

The aim was to test the hypothesis that inhibition of the activation of the Akt and Erk pathways would decrease follicle growth and oestradiol production by ovine ovarian follicles *in vivo*. The oestrous cycles of eighteen ewes were synchronised using a progestagen sponge (Chronogest, Intervet, Boxmeer, The Netherlands) and on Day 3 of the oestrous cycle (oestrus was detected using a raddled vasectomised ram) the two largest follicles were identified (via laparotomy under local anaesthesia), measured, follicular fluid sampled (about 10% of the volume, 4 to 7 μl using a 32G needle) and all other follicles ablated (aspirated and cauterized [27]). This stage of the cycle was chosen as it is during the first follicle wave and at a time when the follicles are large enough to treat but also early enough that the follicles are still growing and producing oestradiol. In each animal the largest of the two remaining follicles was treated (below) and the second follicle served as an untreated control follicle. Ewes were assigned to one of four groups and the largest follicle treated with control medium (n = 4; follicle injected with culture medium plus DMSO), Akt inhibitor (n = 5; follicle injected with LY294002 in control medium), Erk inhibitor (n = 5; follicle injected with PD98059 in control medium) or Akt + Erk inhibitor (n = 4; follicle injected with LY294002 and PD98059 in control medium). The volume of each treatment injection was about 10% of follicle volume (4 to 7 μl), which resulted in a final follicular fluid concentration of 50 μM of the inhibitors, and 50 μM (0.005%) of the DMSO. Concentrations of the inhibitors were based on the treatments used *in vitro* in Experiment 2.

The ewes recovered from surgery and 48 h after treatment (day 5 of the cycle) were euthanized, the two follicles were identified from drawings of the ovaries made at surgery and dissected out of the ovaries, measured and follicular fluid was aspirated. The follicles were cut open and the theca and adherent granulosa cells peeled from the stroma. The granulosa cells were then gently scraped from the theca and the granulosa and theca cells were snap frozen in liquid nitrogen and stored at -80°C [28]. All experimental procedures involving live animals were sanctioned by the UCD Animal Research Ethics Committee and licensed by the Department of Health and Children, Ireland, in accordance with the cruelty to animals act (Ireland, 1987) and European Community Directive 86/609/EC.

### Immunoassays

Inhibin-A concentrations were measured by a two-site IRMA described by Knight and Muttukrishna (1994) [29] which has a detection limit of 250 pg/ml. Oestradiol concentrations were determined by RIA as described previously [23] with a detection limit of 1.5 pg/ml. Progesterone concentrations were determined using an ELISA [30] with a detection limit of 20 pg/ml. Concentrations of both activin-A and follistatin were measured using ELISA [31]. The inter- and intra- assay coefficients for all assays were under 11%.

### Whole cell protein extract preparation

Tissue samples were thawed on ice, homogenised in cold RIPA (Radio-Immunoprecipitation Assay) buffer (50 mM Tris-HCl pH 7.4, 1% NP-40, 150 mM NaCl, 1 mM EDTA, 1 mM PMSF, 1 mM Na_3_VO_4_, 1 mM NaF, 1% protease inhibitor cocktail; P8340, Sigma, Tallaght, Dublin, Ireland) and agitated on a shaker for 15 mins at 4°C. The homogenate was then centrifuged at 1400 rpm for 15 mins at 4°C. The resultant supernatant was snap frozen in liquid nitrogen and stored at -80°C. Protein concentrations of the sample extracts were determined by spectrophotometric assay using the Bio Rad protein assay dye reagent concentrate (Bio Rad Laboratories, #500-0006, Fannin Healthcare, Dublin, Ireland).

### Immunoblotting

Levels of Akt and Erk and their phosphorylated forms were determined as we have previously described [22]. Proteins from granulosa were resolved on 10% SDS polyacrylamide gels (5 μg total protein per sample) and then electrophoretically transferred onto nitrocellulose (Protran^®^, Whatman Schleicher & Schuell Bioscience, Lennox Laboratory Supplies Ltd. Dublin 12, Ireland). The protein transfer was performed at 200 V for 1.5 h at 4°C. Ponceau S (Sigma) stain solution was used to visually assess the equal transfer of the proteins from the gel to the membrane. TBS-Tween was used to destain the membrane, which was then blocked in 5% Marvel in TBS-Tween for 1–2 h. The blocking solution was removed with a brief rinse of TBS-Tween and the membrane was incubated overnight for 14–16 h with the appropriate antibody diluted in 5% BSA in TBS-Tween at 4°C. The antibodies (anti-Akt, anti-phospho-Akt, anti-Erk and anti-phospho-Erk) were all rabbit anti-mouse IgG (New England BioLabs, ISIS, Boghall Road, Bray, Co. Wicklow, Ireland). After incubation with the primary antibody, the membrane was washed twice for 10 min in TBS-Tween and then incubated for a further 1.5 h at room temperature with a polyclonal goat anti-rabbit IgG-HRP conjugated immunoglobulin diluted in 5% Marvel in TBS-Tween (Dako, Cambridge, UK). The secondary antibody was removed and the blot was washed 5 times each for 7 min in TBS-Tween. Protein bands were detected using enhanced chemiluminescence (Supersignal West Femto Max Sensitivity Substrate, Pierce, -Medical Supply Company Ltd., Damastown, Mulhuddart, Dublin 15, Ireland) according to manufacturer's instructions and using autoradiography. Auto-radiographic images of the blots were scanned and the relative intensity (giving a value of 0 for white, no intensity and a value of 256 for black, maximum intensity) of the protein bands was measured using Scion Image software . Background intensity, measured as intensity of area adjacent to selected band, was subtracted from individual values. Within experiments, samples from all treatments were included in each blot to prevent blot-to-blot bias.

### Statistical analysis

In Experiments 1 and 2, hormone concentration and cell number data were analysed by analysis of variance using GLM procedures of SAS and differences between individual treatments were assessed using Tukey's HSD. All values are given as the mean ± SEM.

In Experiment 3, follicular fluid oestradiol concentrations and diameters of treated follicles (largest follicles) and control follicles (second largest follicles) were compared from before treatment to after treatment using a paired Student's *t*-test. Analysis of variance using the GLM procedures of SAS was used to determine the effects of treatment on the levels of Akt, p-Akt, Erk and p-Erk in granulosa and theca cells. All values are given as the mean ± SEM.

## Results

### Experiment 1

#### Effects of FSH and IGF on hormone secretion, cell number and levels of Akt and Erk in granulosa cells *in vitro*

**Figure 1:**
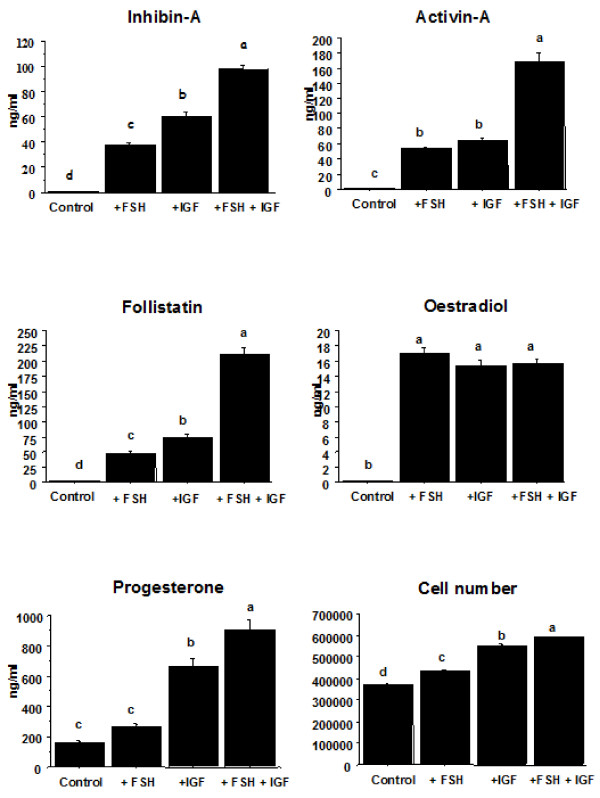
**Effect of treating bovine granulosa cells in vitro with FSH (0.33 ng/ml), IGF-I (10 ng/ml) or FSH plus IGF-I on cell number and secretion of oestradiol, progesterone, inhibin-A, activin-A and follistatin.** Treatment effects were highly significant (P < 0.0001) in all cases (4 replicates with 6 wells included per treatment per replicate). Bars with no common superscript are different (P < 0.05).

Cells treated with FSH or IGF alone showed an increase (P < 0.0001) in the secretion of inhibin-A, activin-A, follistatin and oestradiol, and cell numbers over basal levels (Figure 1). Progesterone secretion was unaffected by FSH treatment alone but was increased (P < 0.01) from cells treated with IGF alone (Figure 1). Co-treatment of granulosa cells with FSH and IGF resulted in enhanced (P < 0.05) secretion of inhibin-A, activin-A, follistatin and progesterone and cell number over and above those from cells treated with either compound alone. In contrast, oestradiol secretion from granulosa cells treated with FSH and IGF in combination was similar (P > 0.05) to that from cells treated with FSH or IGF alone (Figure 1).

**Figure 2:**
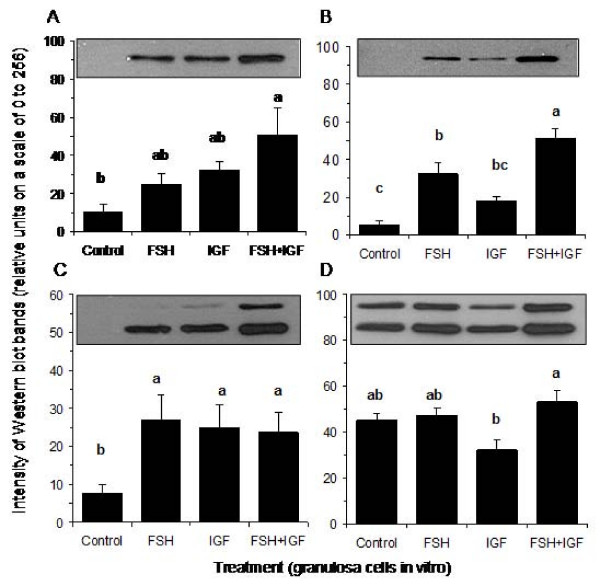
**Representative Western blots and mean levels (± S.E.M) of (A) Akt, (B) p-Akt, (C) Erk and (D) p-Erk in granulosa cells (n = 4) treated with control medium, FSH (0.33 ng/ml), IGF (10 ng/ml) or FSH+IGF in combination in vitro.** Bars with no common superscript are different (P < 0.05). The units represent the intensity of bands after background subtraction and are relative to white (value 0) and black (value 256). The blots each show a single band for Akt and p-Akt at about 60 kDa and each show a double band for Erk and p-Erk at about 44 and 42 kDa.

Only FSH plus IGF in combination stimulated an increase in the levels of total Akt (P < 0.05) compared to the control (Figure 2). Treatment with FSH produced an increase in phospho-Akt compared to control but FSH plus IGF induced an even greater increase in phospho-Akt than FSH alone (P < 0.05) (Figure 2). All treatments increased total Erk levels compared to the control (P < 0.05) with no differences between treatments (Figure 2). Levels of phospho-Erk were similar among all groups except levels were lower in the IGF than the FSH+IGF treatment groups (P < 0.05; Figure 2).

### Experiment 2

#### Effects of inhibition of the Akt and Erk signalling pathways on FSH and IGF action on granulosa cells

**Figure 3:**
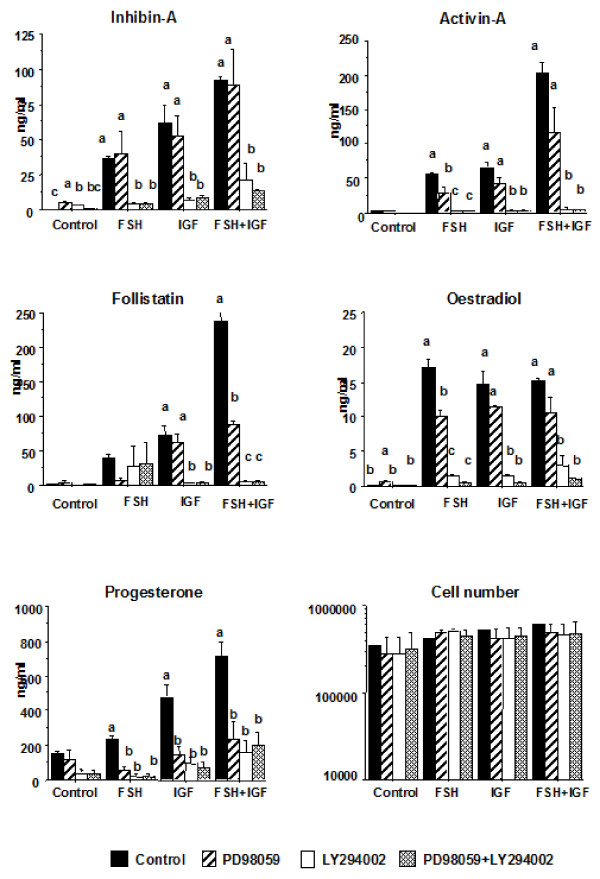
**Effect of treating granulosa cells in vitro with control medium, FSH (0.33 ng/ml), IGF (10 ng/ml) or FSH+IGF in combination with PD98059 (Erk inhibitor) and/or LY2924002 (Akt inhibitor) on cell number and the secretion of oestradiol, progesterone, inhibin-A activin-A and follistatin (N = 3 replicates with 6 wells included per treatment per replicate).** Bars with no common superscript are different (P < 0.05) within each treatment group.

The stimulatory effects of FSH, IGF or their combination were similar to that seen in experiment 1 (Figure 3). Inhibition of the Erk pathway with PD98059 treatment suppressed (P < 0.05) the FSH-induced increase in activin-A, oestradiol and progesterone secretion (Figure 3). Furthermore, PD98059 suppressed follistatin secretion from cells co-stimulated with FSH and IGF and progesterone secretion from cells treated with IGF alone or in combination with FSH. No effect of PD98059 was seen on either FSH or IGF stimulated inhibin-A secretion or viable cell number.

Inhibition of the Akt pathway with LY294002 dramatically reduced (P < 0.05) FSH, IGF or FSH and IGF stimulated inhibin-A, activin-A, oestradiol and progesterone secretion (Figure 3). Follistatin secretion was suppressed in cells treated with IGF alone or in combination with FSH by LY294002 compared to their respective control treatments without LY294002 (Figure 3).

### Experiment 3

#### Effects of LH in combination with PD98059 and/or LY294002 on cell number and secretion of androstenedione and progesterone from theca cells

**Figure 4:**
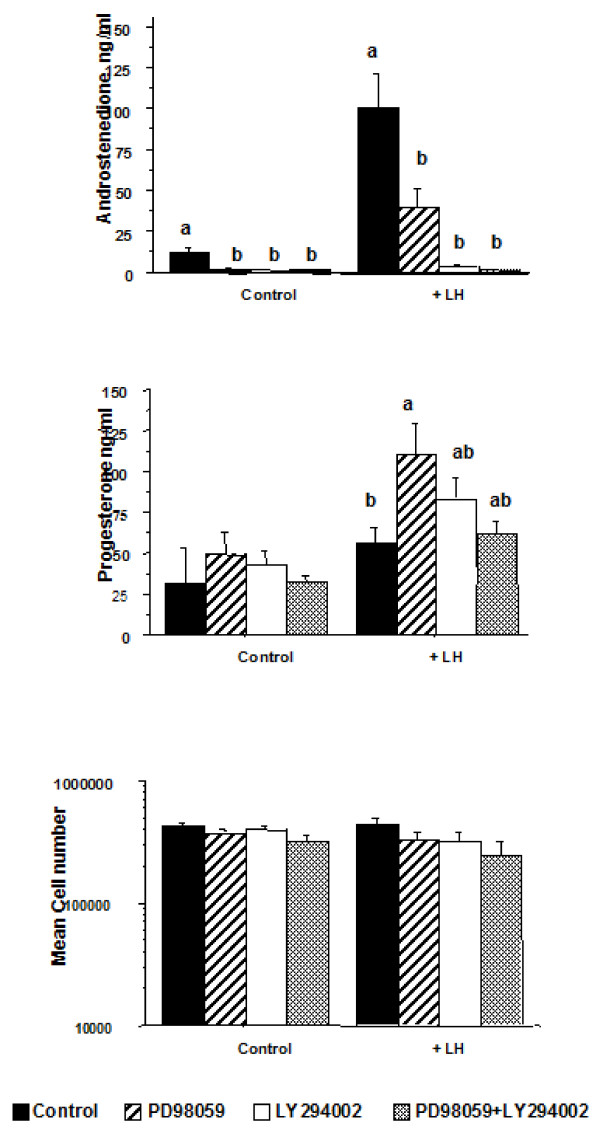
**Effects of treating bovine theca cells in vitro with control medium or LH (160 pg/ml) in combination with PD98059 (Erk inhibitor) and/or LY294002 (Akt inhibitor) on cell number and secretion of androstenedione and progesterone (n = 4 replicates).** Bars with no common superscript are different (P < 0.05) within each treatment group.

Theca cells stimulated with LH showed an 8-fold increase (P < 0.01) in androstenedione secretion compared to the control treatment (Figure 4). Inhibition of the Erk pathway with PD98059 treatment and the Akt pathway with LY294002 reduced (P < 0.05) both basal and LH-induced androstenedione secretion compared to controls (Figure 4). Progesterone concentrations in media were not affected (P > 0.05) by LH stimulation but treatment with PD98059+LH stimulated an increase in progesterone concentrations compared to LH alone (Figure 4). Neither the Erk nor Akt inhibitors affected the number of viable theca cells at the end of culture (P > 0.05).

### Experiment 4

**Figure 5:**
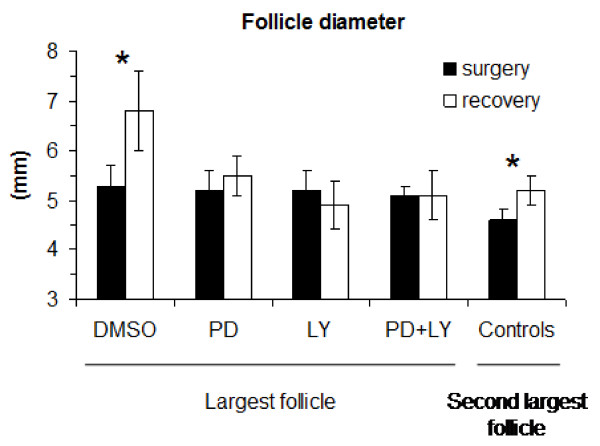
**Follicle diameter (mean ± sem) in ewes in which the largest follicle was treated in vivo with control solution with DMSO (DMSO n = 5), PD98059 (PD, n = 5), LY294002 (LY, n = 4) or PD98059 plus LY294002 (PD+LY, n = 4).** The second follicle in each animal served as an untreated control control (n = 18). All other follicles were ablated via electrocautery. Follicle diameter was measured at the time of surgery (via laparotomy) and 48 h later after the ovaries were recovered. * indicates differences (P < 0.05) between diameters at surgery and recovery.

**Figure 6:**
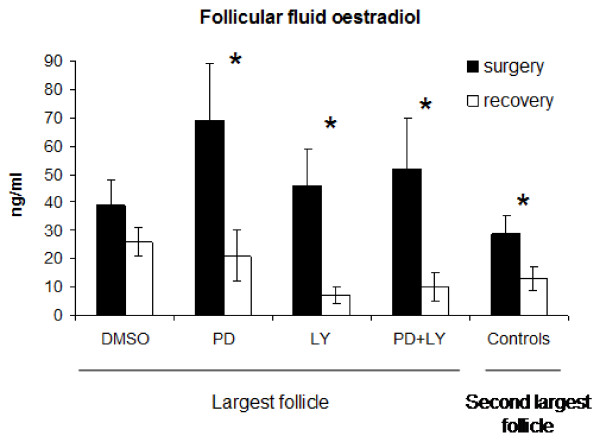
**Follicular fluid oestradiol concentrations (mean ± sem) in ewes in which the largest follicle was treated in vivo with control solution with DMSO (DMSO n = 5), PD98059 (PD, n = 5), LY294002 (LY, n = 4) or PD98059 plus LY294002 (PD+LY, n = 4).** The second follicle in each animal served as an untreated control (n = 18). All other follicles were ablated via electrocautery. Follicular fluid was sampled from follicles at the time of surgery (via laparotomy) and 48 h later after the ovaries were recovered. * indicates differences (P < 0.05) between concentrations at surgery and recovery.

Follicle diameters and follicular fluid oestradiol concentrations were not different (P > 0.05) among groups for the largest (subsequently treated) follicles or the second largest (control) follicles before treatment (Figures. 5 and 6). However, both the diameter (5.2 ± 0.2 vs 4.6 ± 0.2 mm; combined means; P = 0.0001) and follicular fluid oestradiol concentrations (51.3 ± 7.7 vs 29.4 ± 6.2 ng/ml; P = 0.018) where greater in the largest compared to the second largest follicles before treatment.

Of the treated follicles, only the control follicles that were treated with DMSO increased in diameter (P = 0.029) between the time of injection and 48 h later when recovered (Figure 5). The other follicles treated with PD98059, LY294002 or PD98059 plus LY294002 showed no increase (P > 0.05) in diameter over the same period (Figure 5). The untreated, second largest, control follicles also increased in diameter (P = 0.03; Figure 5). Follicular fluid oestradiol concentrations were similar between the time of injection (at surgery) and recovery of the ovaries 48 h later in the control follicles treated with DMSO (P > 0.05) but decreased in follicles treated with PD98059 (P = 0.02), LY294002 (P = 0.01) and PD98059+LY294002 (P = 0.05). Follicular fluid oestradiol concentrations also decreased (P < 0.05) in the second largest (control) follicles over the 48 h period (Figure 6).

## Discussion

Findings from the present study indicate that inhibition of the Akt and Erk pathways inhibit the stimulatory actions of FSH and IGF on cultured bovine granulosa cells and LH on theca cells *in vitro*. Furthermore, inhibition of the Akt and Erk pathways *in vivo* had a negative effect on follicular fluid oestradiol production and follicle growth in sheep. Taken together, these results suggest an important role for Akt and Erk signalling pathways in mediating the effects of the gonadotropins and IGF on follicle cell function and on follicular development.

The stimulation of inhibin-A, activin-A, follistatin, oestradiol, progesterone and cell number by FSH and IGF in granulosa cells *in vitro* agrees with earlier findings [23]. However, the regulation of the Akt and Erk pathways in relation to these hormonal and proliferative changes has not been studied previously in the bovine model. Increases in Akt and Erk signalling proteins in response to FSH and IGF stimulation suggest a role for Akt and Erk signal transduction pathways in FSH and IGF mediated granulosa cell development as reflected by cell proliferation/survival and production of inhibin-A, activin-A, follistatin, oestradiol, and progesterone (Figure 1). The significant reductions in hormonal output as a result of inhibition of the Akt and Erk pathways further support a role for Akt and Erk in FSH- and IGF- mediated action in granulosa cells. However, there appear to be differences in the relative importance of each pathway with respect to the endpoints measured. Our findings suggest that Akt is important in mediating the effects of FSH on inhibin-A, activin-A, oestradiol and progesterone secretion and also important in mediating IGF-I stimulated inhibin-A, activin-A, follistatin, oestradiol and progesterone secretion by granulosa cells. In addition, the results also suggest that the Erk pathway is involved in mediating FSH-induced activin-A and oestradiol production, and progesterone secretion induced by both FSH and IGF-I stimulation of granulosa cells *in vitro*.

The regulation of activin-A secretion by FSH and IGF displayed a similar pattern to that of oestradiol with the Erk pathway only involved in FSH-stimulated production and the Akt pathway involved in both FSH- and IGF-stimulated production. Inhibition of the Erk pathway had no effect on inhibin-A concentrations. Only the Akt pathway was indicated in regulating the production of inhibin-A. However, this might be a simplistic view of what is happening. Activin is known to upregulate FSH receptors and aromatase gene expression, thus promoting production of oestradiol [32]. Additionally, expression of the inhibin α-subunit is increased in response to activin-A [33]. Previous work suggests that activin-A may mediate the effects of FSH stimulation on oestradiol and inhibin-A production [23] but this explanation remains to be proved. The observed differences in oestradiol and inhibin-A production in this present study might not relate directly to inhibition of the Akt and Erk pathways but rather the indirect effect of inhibition of these pathways on regulation of activin-A production/secretion.

Granulosa cell proliferation is a critical step in follicular development and both FSH and IGF are required for successful follicle development. Our results (Figure 1) confirmed other research showing that FSH and IGF promote proliferation/survival of granulosa cells [5,34]. Despite the fact that FSH and IGF stimulated the Akt and Erk pathways (Figure 2) and that inhibition of these pathways markedly influenced hormone secretion, neither inhibitor affected FSH and IGF stimulated increases in cell number (Figure 3). It may be that additional signalling pathways activated by FSH and IGF, such as PKA [15], compensated for the block in Akt and Erk signalling. Our findings are not in agreement with others that found that FSH-stimulated porcine granulosa cell proliferation/survival was significantly reduced by treatment with PD98059 through a negative effect on cell cycle proteins and DNA synthesis [9,35,36].

In addition to FSH and IGF, LH is also important for follicle development and it has been shown that LH increases activation of Erk Akt in porcine and rat theca cells [10,12]. As expected from previous studies on bovine theca cells [37], our results demonstrated a marked increase in androstenedione production by theca cells in response to LH (Figure 4). Moreover, this LH-induced increase was attenuated by inhibition of Erk and completely blocked by inhibition of the Akt pathway. Conversely, progesterone production increased in response to inhibition of the Erk pathway. This is in agreement with other recent findings that demonstrated that LH-induced Erk activation differentially regulates production of progesterone and androstenedione in bovine theca cells *in vitro*[38].

The results from Experiment 4 clearly indicate that treatment of follicles *in vivo* with inhibiters of the Akt and Erk pathways in the largest follicle in sheep had a negative effect on follicular oestradiol production and follicle growth, two key markers of follicle health and dominant follicle development. There was a difference between the largest and second largest follicles at the start of treatment with respect to diameter and oestradiol concentration, which agrees with previous findings that showed that ovine follicles exist in a hierarchy in relation to follicle diameter and oestradiol concentrations [27]. Day 3 of the cycle was chosen as the day of treatment in the present study as follicles would be large enough to treat, be producing relatively high amounts of oestradiol and still be growing. Previous research indicated that between Days 1 and 3 of the cycle oestradiol concentrations increase; however, that they then start to decline on Day 4 [39]. None-the-less, despite treating follicles relatively late in the follicle wave we still demonstrated an inhibitory effect on follicle growth and oestradiol production through blocking the activation of Akt and Erk pathways.

The significant decrease in oestradiol concentrations in follicles treated *in vivo* with Akt and Erk inhibitors agrees with the results from Experiments 1 and 2 where inhibition of the Erk pathway inhibited FSH-induced oestradiol production and inhibition of the Akt pathways inhibited both FSH- and IGF-induced oestradiol production in granulosa cells *in vitro *(Figure 3). Androstenedione secretion in cultured theca cells was also abrogated by inhibition of both the Akt and Erk pathways (Figure 4). In Experiment 3, the inhibitors were injected directly into the antral cavity and it is reasonable to suggest that granulosa cells would be first to be exposed to and affected by the inhibitors. However, it is possible that the inhibitors might have diffused through the granulosa layer of cells into the theca layer and affect signalling pathways there. Thus the significant reductions in follicular fluid oestradiol concentrations may be due to the effect of the Akt and Erk inhibitors on both granulosa and theca cells in combination.

In summary, this study demonstrates a role for the Akt and Erk pathways in mediating the actions of FSH and IGF on granulosa cells and LH on theca cells *in vitro* and their role in follicle growth and oestradiol secretion *in vivo*. While both pathways appear to be important for the actions of these hormones in both cell types, we conclude that the actions of the Akt pathway are more pronounced than the Erk pathway in granulosa cells and vice versa in the in theca cells. None the less, administration of inhibitors of these pathways *in vivo* inhibited follicle growth and reduced follicular fluid oestradiol concentrations. We suggest that the successful functioning of healthy follicles requires the activation of the Akt and Erk signal transduction pathways, and that these pathways are necessary for ovarian follicle growth and development.

## Competing interests

The authors declare that they have no competing interests.

## Authors' contributions

KR was responsible for coordinating and conducting all the tasks for the study in collaboration with co-authors. CG and PK participated in cell culture, and immunoassays. AE and PL participated in the *in vivo* parts of the project. All authors contributed to experimental design, data analysis and interpretation, and have read and approved the final manuscript.

## References

[B1] Evans ACO (2003). Characteristics of ovarian follicle development in domestic animals. Reproduction in Domestic Animals.

[B2] Gibbons JR, Kot K, Thomas DL, Wiltbank MC, Ginther OJ (1999). Follicular and FSH dynamics in ewes with a history of high and low ovulation rates. Theriogenology.

[B3] Bartlewski PM, Beard AP, Rawlings NC (2000). An ultrasound-aided study of temporal relationships between the patterns of lh/fsh secretion, development of ovulatory-sized antral follicles and formation of corpora lutea in ewes. Theriogenology.

[B4] Adams GP, Matteri RL, Katelic JP, Ko JCH, Ginther OJ (1992). Association between surges of follicle-stimulating hormone and the emergence of follicular waves in heifers. J Reprod Fertil.

[B5] Robker RL, Richards JS (1998). Hormone-Induced proliferation and differentiation of granulosa cells: a coordinated balance of the cell cycle regulators cyclin D2 and p27Kip1. Molecular Endocrinology.

[B6] Austin EJ, Mihm M, Evans ACO, Knight PG, Ireland JLH, Ireland JJ, Roche JF (2001). Alterations in intrafollicular regulatory factors and apoptosis during selection of follicles in the first follicular wave of the bovine estrous cycle. Biology of Reproduction.

[B7] Campbell BK, Dobson H, Baird DT, Scaramuzzi RJ (1999). Examination of the relative role of FSH and LH in the mechanism of ovulatory follicle selection in sheep. Journal of Reproduction and Fertility.

[B8] Seger R, Hanoch T, Rosenberg R, Dantes A, Merz WE, Strauss JF, Amsterdam A (2001). The ERK signaling cascade inhibits gonadotropin-stimulated steroidogenesis. Journal of Biological Chemistry.

[B9] Babu PS, Krishnamurthy H, Chedrese PJ, Sairam MR (2000). Activation of extracellular-regulated kinase pathways in ovarian granulosa cells by the novel growth factor type 1 follicle-stimulating hormone receptor. Role in hormone signaling and cell proliferation. Journal of Biological Chemistry.

[B10] Cameron MR, Foster JS, Bukovsky A, Wimalasena J (1996). Activation of mitogen-activated protein kinases by gonadotropins and cyclic adenosine 5'-monophosphates in porcine granulosa cells. Biology of Reproduction.

[B11] Zeleznik AJ, Saxena D, Little-Ihrig L (2003). Protein kinase B is obligatory for follicle-stimulating hormone-induced granulosa cell differentiation. Endocrinology.

[B12] Carvalho CR, Carvalheira JB, Lima MH, Zimmerman SF, Caperuto LC, Amanso A, Gasparetti AL, Meneghetti V, Zimmerman LF, Velloso LA, Saad MJ (2003). Novel signal transduction pathway for luteinizing hormone and its interaction with insulin: activation of Janus kinase/signal transducer and activator of transcription and phosphoinositol 3-kinase/Akt pathways. Endocrinology.

[B13] Richards JS, Fitzpatrick S, Clemens JW, Morris JK, Alliston T, Sirois J (1995). Ovarian cell differentiation: a cascade of multiple hormones, cellular signals, and regulated genes. Recent Progress in Hormone Research.

[B14] Pennybacker M, Herman B (1991). Follicle-stimulating hormone increases c-fos mRNA levels in rat granulosa cells via a protein kinase C-dependent mechanism. Molecular and Cellular Endocrinology.

[B15] Hunzicker-Dunn M, Maizels ET (2006). FSH signaling pathways in immature granulosa cells that regulate target gene expression: branching out from protein kinase A. Cell Signal.

[B16] Davidson TR, Chamberlain CS, Bridges TS, Spicer LJ (2002). Effect of follicle size on in vitro production of steroids and insulin-like growth factor (IGF)-I, IGF-II, and the IGF-binding proteins by equine ovarian granulosa cells. Biology of Reproduction.

[B17] Echternkamp SE, Howard HJ, Roberts AJ, Grizzle J, Wise T (1994). Relationships among concentrations of steroids, insulin-like growth factor-I, and insulin-like growth factor binding proteins in ovarian follicular fluid of beef cattle. Biology of Reproduction.

[B18] Fortune JE, Rivera GM, Yang MY (2004). Follicular development: the role of the follicular microenvironment in selection of the dominant follicle. Animal Reproduction Science.

[B19] Chamoun D, Choi D, Tavares AB, Udoff LC, Levitas E, Resnick CE, Rosenfeld RG, Adashi EY (2002). Regulation of granulosa cell-derived insulin-like growth factor binding proteins (IGFBPs): role for protein kinase-C in the pre- and posttranslational modulation of IGFBP-4 and IGFBP-5. Biology of Reproduction.

[B20] Jenkins PJ, Bustin SA (2006). Evidence for a link between IGF-I and cancer. European Journal of Endocrinology.

[B21] Evans ACO, Martin F (2000). Kinase pathways in dominant and subordinate ovarian follicles during the first wave of follicular development in sheep. Animal Reproduction Science.

[B22] Ryan KE, Casey SM, Canty MJ, Crowe MA, Martin F, Evans ACO (2007). Akt and Erk signal transduction pathways are early markers of differentiation in dominant and subordinate ovarian follicles in cattle. Reproduction.

[B23] Glister C, Tannetta DS, Groome NP, Knight PG (2001). Interactions between follicle-stimulating hormone and growth factors in modulating secretion of steroids and inhibin-related peptides by nonluteinized bovine granulosa cells. Biology of Reproduction.

[B24] Gutierrez, Campbell, Webb R (1997). Development of a long-term bovine granulosa cell culture system: induction and maintenance of estradiol production, response to follicle-stimulating hormone, and morphological characteristics. Biology of Reproduction.

[B25] Dudley DT, Pang L, Decker SJ, Bridges AJ, Saltiel AR (1995). A synthetic inhibitor of the mitogen-activated protein kinase cascade. Proceedings of the National Academy of Sciences USA.

[B26] Vlahos CJ, Matter WF, Hui KY, Brown RF (1994). A specific inhibitor of phosphatidylinositol 3-kinase, 2-(4- morpholinyl)-8-phenyl-4H-1-benzopyran-4-one (LY294002). Journal of Biological Chemistry.

[B27] Evans ACO, Flynn JD, Duffy P, Knight PG, Boland MP (2002). Effects of ovarian follicle ablation on FSH, oestradiol and inhibin A concentrations and growth of other follicles in sheep. Reproduction.

[B28] Evans ACO, Fortune JE (1997). Selection of the dominant follicle in cattle occurs in the absence of differences in the expression of messenger ribonucleic acid for gonadotropin receptors. Endocrinology.

[B29] Knight PG, Muttukrishna S (1994). Measurement of dimeric inhibin using a modified two-site immunoradiometric assay specific for oxidized (Met O) inhibin. Journal of Endocrinology.

[B30] Sauer MJ, Foulkes JA, Worsfold A, Morris BA (1986). Use of progesterone 11-glucuronide-alkaline phosphatase conjugate in a sensitive microtitre-plate enzymeimmunoassay of progesterone in milk and its application to pregnancy testing in dairy cattle. J Reprod Fertil.

[B31] Tannetta DS, Feist SA, Bleach ECL, Groome NP, Evans LW, Knight PG (1998). Effects of active immunization of sheep against an amino terminal peptide of the inhibin αC subunit on intrafollicular levels of activin A, inhibin A and follistatin. Journal of Endocrinology.

[B32] Nakamura M, Minegishi T, Hasegawa Y, Nakamura K, Igarashi S, Ito I, Shinozaki H, Miyamoto K, Eto Y, Ibuki Y (1993). Effect of an activin A on follicle-stimulating hormone (FSH) receptor messenger ribonucleic acid levels and FSH receptor expressions in cultured rat granulosa cells. Endocrinology.

[B33] Findlay JK (1993). An update on the roles of inhibin, activin, and follistatin as local regulators of folliculogenesis. Biology of Reproduction.

[B34] Monget P, Fabre S, Mulsant P, Lecerf F, Elsen J-M, Mazerbourg S, Pisselet C, Monniaux D (2002). Regulation of ovarian folliculogenesis by IGF and BMP system in domestic animals. Domestic Animal Endocrinology.

[B35] Shiota M, Sugai N, Tamura M, Yamaguchi R, Fukushima N, Miyano T, Miyazaki H (2003). Correlation of mitogen-activated protein kinase activities with cell survival and apoptosis in porcine granulosa cells. Zoological Science.

[B36] Kayampilly PP, Menon KMJ (2004). Inhibition of extracellular signal-regulated protein kinase-2 phosphorylation by dihydrotestosterone reduces follicle-stimulating hormone-mediated cyclin D2 messenger ribonucleic acid expression in rat granulosa cells. Endocrinology.

[B37] Glister C, Richards SL, Knight PG (2005). Bone morphogenetic proteins (BMP -4, -6 and -7 potently suppress basal and luteinizing hormone-induced androgen production by bovine theca interna cells in primary culture: could ovarian hyperandrogenic dysfunction be caused by a defect in thecal BMP signaling?. Endocrinology.

[B38] Tajima K, Yoshii K, Fukuda S, Orisaka M, Miyamoto K, Amsterdam A, Kotsuji F (2005). LH-induced ERK activation differently modulates progesterone and androstenedione production in bovine theca cells. Endocrinology.

[B39] Souza CJH, Campbell BK, Baird DT (1997). Follicular dynamics and ovarian steroid secretion in sheep during the follicular and early luteal phases of the estrous cycle. Biology of Reproduction.

